# DNA Methylation Mediates the Transcription of *STAT4* to Regulate *KISS1* During Follicular Development

**DOI:** 10.3390/cells14070523

**Published:** 2025-04-01

**Authors:** Danxia Chen, Ming Fang, Enyuan Huang, Hongyan Quan, Liuhong Zhang, Yingting He, Xiaofeng Zhou, Bin Ma, Xiaolong Yuan, Jiaqi Li

**Affiliations:** 1 State Key Laboratory of Swine and Poultry Breeding Industry, National Engineering Research Center for Breeding Swine Industry, Guangdong Provincial Key Laboratory of Agro-Animal Genomics and Molecular Breeding, College of Animal Science, South China Agricultural University, Guangzhou 510642, China; danxia0218@163.com (D.C.); fming@stu.scau.edu.cn (M.F.); heeeyy@stu.scau.edu.cn (E.H.); qhy@stu.scau.edu.cn (H.Q.); zhanglh@stu.scau.edu.cn (L.Z.); he_yingting@163.com (Y.H.); zxf@scau.edu.cn (X.Z.); 2 Centre for Healthy Ageing, Health Futures Institute, Murdoch University, Murdoch, WA 6150, Australia; b.ma@murdoch.edu.au; 3 National Center of Technology Innovation for Pigs, Chongqing 402460, China

**Keywords:** *STAT4*, DNA methylation, GCs, follicle, mammals

## Abstract

Maturation of follicles is the primary condition for the initiation of puberty, and excessive apoptosis of granulosa cells (GCs) will hinder the normal development of follicles in pigs. Signal Transducer and Activator of Transcription 4 (*STAT4*) plays an important role in cell proliferation and apoptosis. However, the mechanism of DNA methylation regulating *STAT4* transcription and affecting follicle development in pigs remains unclear. To resolve this problem, we constructed a *STAT4* overexpression vector and interference fragment to explore the effects of *STAT4* on GC function and investigate the effects of changes in methylation status of the *STAT4* promoter region on cell function and kisspeptin-1 (*KISS1*) expression, as well as the *STAT4* effects on the development of the follicles of pigs and mice in vitro. We found that the expression of *STAT4* decreased, while DNA methylation of the *STAT4* promoter region increased with the growth of the follicles. After overexpression of *STAT4*, the apoptosis of GCs was increased but the proliferation, cell cycle and estrogen secretion of GCs were inhibited. When GCs were treated with DNA methyltransferase inhibitor (5-Aza-CdR), the methylation of the *STAT4* promoter region decreased, resulting in a significant increase in the expression of *STAT4*. Consequently, the expression of *KISS1* was inhibited. At the same time, the expressions of genes related to cell proliferation, cell cycle and estrogen secretion signaling pathways decreased, while the expressions of genes related to the apoptosis signaling pathway increased. After infection with the *STAT4* lentiviral vector (LV-*STAT4*) in follicles of mice, the expression of *STAT4* in ovaries of mice significantly increased, and the expression of *KISS1* was significantly decreased. The capillaries on the surface of follicles were constricted, the age of puberty onset in mice was delayed while the levels of GnRH, LH, FSH and E2 in serum were decreased. In conclusion, we found that reduced methylation status of the *STAT4* promoter region promoted the transcription of *STAT4* and then inhibited the expression of *KISS1*, as well as promoted the apoptosis of GCs and ultimately inhibited the normal development of follicles in mammals.

## 1. Introduction

The development and maturation of ovarian follicles are essential conditions for the initiation of puberty in mammals [1,2,3]. However, about 99% of follicles become atretic during follicular growth, with only about 1% reaching full maturation [4,5,6]. This low maturation rate significantly impacts reproductive efficiency in pigs [7]. Follicular development is influenced by multiple factors, including insulin-like growth factors [8] and epigenetic regulation [9]. For instance, the accumulation of the protein p53 induces apoptosis of granulosa cells (GCs) and follicular atresia by upregulating the Fas/Fas ligand [10]. Additionally, aquaporins participate in the estrogen-mediated regulation of follicular development by modulating cell cycle progression and apoptosis of GCs in buffalo follicles [11]. Overexpression of *FOXO3* has been shown to induce the apoptosis of GCs, leading to follicular atresia in porcine follicles [12]. In summary, follicular atresia is closely associated with the apoptosis of GCs, thereby influencing follicular development in pigs. Nevertheless, the regulatory mechanisms underlying follicular atresia mediated by the apoptosis of GCs in pigs remain unclear.

DNA methylation, an epigenetic chemical modification, affects gene expression by altering the methylation levels around the promoter [13,14,15]. Previous studies have demonstrated that DNA methylation plays a role in follicle development in mammals [16,17,18]. For instance, the DNA methylation of promoter regions is negatively correlated with lncRNA expression during puberty in goats [19]. In mammalian follicles, *SLCO3A1* enhances the proliferation of GCs and promotes follicle development through the knockdown of *DNMT1* [20]. Furthermore, DNA methylation may influence the PI3K-AKT signaling pathway, the GnRH signaling pathway and the secretion of estradiol (E2) during puberty in gilts [21]. In addition, demethylation of the *RSPO2* promoter facilitates proliferation and inhibits the apoptosis of GCs by upregulating *RSPO2* expression in pigs [22]. The methylation states of the inhibitory factor of follicular development (*IFFD*) are regulated by *DNMT1*, which suppresses proliferation of GCs and E2 secretion while promoting the apoptosis of GCs [23]. Above all, alterations in the DNA methylation of key genes in GCs are likely to play a crucial role in follicle development and puberty in mammals.

The Signal Transducer and Activator of Transcription (STAT) family of proteins plays a critical role in cellular processes such as apoptosis, proliferation and immune response by interacting with DNA [24]. In adult mare ovaries, the mRNA expression of *STAT3* is significantly upregulated, and the JAK/STAT signaling pathways are actively involved in folliculogenesis [25]. In cattle, follicular atresia due to insufficient follicle-stimulating hormone (FSH) is associated with activated leukemia inhibitory factor (LIF)-*STAT3* signaling in GCs [26]. Additionally, during follicular deviation in bovines, an increased abundance of phosphorylated *STAT3* in GCs indicates its involvement in the apoptosis of GCs and subsequent follicular atresia [27]. In porcine follicular development, CCAAT/enhancer-binding protein beta (C/EBPβ) enhances the anti-apoptotic and pro-proliferative effects of *STAT3* in GCs [28]. Furthermore, *STAT4* has been cloned in porcine tissues [29] and has been shown to facilitate the apoptosis of GCs under hypoxia conditions, thereby blocking follicular development [30]. It is also suggested that *STAT4* may negatively regulate the transcriptional activity and biological functions of *KISS1*, leading to reduced E2 synthesis in GCs and arrested follicular development [31]. IL-11 upregulates the expression of prostaglandin endoperoxide synthase 2 (PTGS2) by activating the JAK1/STAT3 signaling pathway, thereby promoting the production of prostaglandin E2 (PGE2) in bovine GCs [32]. The necroptosis of GCs in premature ovarian failure is triggered by up- and downregulating the reticulophagy receptor CCPG1 through active *STAT1*/*STAT3* [33]. The other research found that miR-520h inhibited the development of polycystic ovary syndrome by targeting IL6R, potentially through activation of the JAK/STAT pathway, thereby regulating granulosa cell proliferation and apoptosis [34]. These studies demonstrated that *STAT4* may regulate the function of GCs, and the activation of the STAT family may be affected by ligands such as IL-11. However, the mechanism by which DNA methylation of the *STAT4* promoter regulates apoptosis of GCs and the onset of puberty in pigs remains to be elucidated.

In this study, we utilized the GCs model to modulate the methylation status of the *STAT4* promoter region using 5-Aza-CdR. Our aim was to explore how changes in *STAT4* expression and promoter methylation status affect the growth and development of follicles. Additionally, we also aimed to elucidate the regulatory mechanism by which *STAT4* modulates the expression of *KISS1*. This involved investigating the impact of altered *STAT4* methylation status on its transcription and subsequently examining how changes in *STAT4* expression influence the development of follicles in mammals.

## 2. Materials and Methods

### 2.1. Animals

Three-week-old female C57BL/6 mice were purchased from South Medical University (Guangzhou, China) for the in vivo experimental study. The mice were allocated into four groups randomly: LV-*STAT4* (*n* = 15), LV-NC (*n* = 10), sh-*STAT4* (*n* = 15) and sh-NC (*n* = 10). Following a three-day acclimatization feeding period in the mouse facility with a temperature of 21~26 °C, the mice were intraperitoneally injected with lentivirus 1 × 10^7^ TU once per week for three consecutive weeks. The lentiviral vectors used for *STAT4* overexpression and knockdown were synthesized by Guangdong Dong ze Biological (Guangzhou, China). The vaginal openings of the mice were observed daily to ascertain their estrous cycle stage.

### 2.2. Culture of GCs and Follicles

The GCs were cultured in Dulbecco’s modified Eagle’s medium (DMEM; Hyclone, Logan, UT, USA) supplemented with 10% fetal bovine serum (FBS; Hyclone, Logan, UT, USA) and 1% penicillin/streptomycin (Invitrogen, Shanghai, China). Fresh, healthy porcine ovaries were obtained from a local slaughterhouse and rinsed twice with pre-chilled phosphate-buffered saline (PBS) containing 1% penicillin/streptomycin. Follicular fluid was aspirated from the follicles using a 1 mL syringe and centrifuged in DMEM. The cells isolated from the follicles were rinsed twice with PBS, resuspended and seeded into 75 cm^2^ culture flasks. The cells were cultivated in culture solution that contained 10% fetal bovine serum and 1% penicillin/streptomycin at 37 °C and in a 5% CO_2_ atmosphere.

For the follicle culture, porcine ovaries were gathered from a local slaughterhouse; then, ovarian follicles (3–4 mm diameter) were isolated after being rinsed twice with PBS. The follicles were then incubated in a 5% CO_2_ atmosphere at 38.5 °C for 24 h to assess their morphology and contamination status. Subsequently, the follicles were subjected to in vitro treatment with either an overexpression or knockdown lentiviral vector targeting *STAT4* (1 × 10⁷ TU). After three days of culture, the follicles were collected, and their status was documented.

### 2.3. Real-Time Quantitative PCR (qRT-PCR)

We extracted total RNA with Trizol reagent (TaKaRa, Tokyo, Japan). The obtained RNA was then reversely transcribed into cDNA with a reverse transcription kit (TaKaRa), following this reaction procedure: 15 min at 37 °C, 5 s at 85 °C and an indefinite hold at 4 °C. qPCR was conducted using Maxima SYBR Green qPCR Master Mix (2×) (YEASEN, Shanghai, China) on the CFX96 Touch Real-Time PCR System (Bio-Rad, Berkeley, CA, USA). GAPDH served as the housekeeping gene, and the 2^−ΔΔCT^ method was employed to determine the relative expression of target mRNAs.

### 2.4. EdU Assay

To evaluate cell proliferation, EdU assays were conducted using the Cell-LightTM EdU Apollo 567 kit (RiboBio, Guangzhou, China). Cells were seeded into 48-well plates and then transfected with either overexpression or interfering plasmids. Following transfection for 48 h, we added 80% acetone into the culture plate for 30 min to fix the cells. The cells were then rinsed twice with PBS and permeabilized using 0.5% Triton X-100 for 10 min. Subsequently, the cells were treated with 1× Apollo for 30 min and then stained with 1× Hoechst for an additional 30 min. The whole process was conducted under conditions that minimized light exposure. The microscopy fluorescence was performed within 48 h to visualize EdU incorporation. The proliferation rate was calculated by determining the ratio of EDU-positive cells to Hoechst-positive cells within the same view.

### 2.5. Cell Counting Kit-8 Viability Assay (CCK-8)

The cell proliferation and viability were assessed using the Cell Counting Kit-8 (BioSharp, Chengdu, China). Cells were seeded into 96-well plates and transfected with either overexpression or interference plasmids. Subsequently, 5 μL of plasmids of the *STAT4* overexpression vector and interference fragment was added to each well to stimulate the cells separately. The CCK8 assay was conducted at 12 h, 24 h, 36 h and 48 h after treatment. The viability of cells was determined by quantifying the absorbance at 450 nm using a microplate reader (IMark; Bio-Rad Laboratories, CA, USA). The measured absorbance at this wavelength is directly correlated with the quantity of viable cells. The formula was as follows:

$$cell\;viability\left(\%\right)=\frac{{OD}_{sample}-{OD}_{black}}{{OD}_{control}-{OD}_{black}}\times 100\%$$

### 2.6. Flow Cytometry

We used the Annexin V-FITC Apoptosis Detection Kit (BioVision, Milpitas, CA, USA) to assess the apoptosis rate. Following culture in 6-well plates, cells were harvested and rinsed twice with PBS. Subsequently, the cells were gently resuspended in 500 μL of 1× Annexin V buffer. Then, we added 5 μL of Annexin V-FITC and 5 μL of PI staining solution to the cells, mixed gently and incubated at room temperature in the dark for 15 min. We used flow cytometry to measure the apoptosis rate of cells, and the data were analyzed using Flowjo software 7.6 (BD, Baltimore, MD, USA).

Otherwise, we used a cell cycle kit (KeyGEN, Nanjing, China) to detect the cycle distribution of cells. The cells were transferred into 15 mL centrifuge tubes and washed with pre-cooled PBS. Following this, they were fixed using 75% ethanol and kept at 4 °C for 4 h. The cells were treated with PI/RNase staining buffer and incubated at 37 °C for 30 min in the dark after fixation. The cell cycle distribution was then examined using flow cytometry.

### 2.7. ELISA

To measure the E2, luteinizing hormone (LH), FSH and gonadotropin-releasing hormone (GnRH) concentrations in this research, an ELISA kit from Jianglai Biological Co., Ltd. (Shanghai, China). was utilized. According to the instructions of the kit, we added the varying concentrations of standard samples to the standard wells, while the supernatant samples were allocated to designated sample wells. Antibody reagent was then added to both the standard and sample wells. The plate was incubated at 37 °C for 1 h. The wells were rinsed with double-distilled water after incubation, and the substrate solution was added subsequently. The plate was then incubated in the dark at 37 °C for 15 min. The reaction was terminated by the addition of a stop solution, then the optical density (OD) was measured at 450 nm.

### 2.8. Hematoxylin and Eosin Staining (HE)

The mouse ovaries and pig follicles were assessed using HE. Firstly, we used 4% paraformaldehyde to fix the tissues and then embedded them in paraffin; then, we sectioned them to obtain the largest cross-sections of the follicles, with each section being 3 μm thick. The paraffin sections were then stained with hematoxylin for 1 min and briefly counterstained with eosin. Following a rinse with running water, the stained sections were examined under a Nikon ECLIPSE Ti2 fluorescence microscope (Nikon, Tokyo, Japan).

### 2.9. Bisulfite Sequencing PCR (BSP)

Genomic DNA was isolated from GCs using a tissue DNA extraction kit (D3396-02, Omega Bio-Tek, Norcross, GA, USA). The purified DNA underwent bisulfite conversion using the EZ DNA Methylation-GoldTM Kit (D5006, ZYMO RESEARCH, Tustin, CA, USA). Bisulfite-specific PCR (BSP) primers were then utilized to amplify the target DNA fragments. The amplified products were cloned into pMD-18T vector (Takara, Kyoto, Japan), and 8 randomly selected clones per group were sequenced. The methylation status of the sequencing results was analyzed by aligning with the original genomic sequences. Data analysis and visualization of methylation patterns were performed using the QUMA online tool (http://quma.cdb.riken.jp/).

### 2.10. Chromatin Immunoprecipitation Assay (ChIP)

The ChIP assay was performed with the Pierce Agarose ChIP Kit (Thermo Scientific, Shanghai, China). The specific operation referred to the instruction manual of the kit, which was roughly divided into cell cross-linking with complete medium containing 1% formaldehyde for 10 min and collection and then terminating with glycine solution. After cross-linking, the cell precipitate was collected, and the granulocytes were lysed and digested with the *STAT4* and *KISS1* antibody for the immunoprecipitation reaction. Finally, the immunoprecipitated complexes were analyzed via PCR, and the results were visualized using agarose gel electrophoresis.

### 2.11. Dual-Luciferase Reporter Gene Assay

The dual-luciferase reporter assay was conducted using the Dual-Luciferase Reporter Assay Kit (Shanghai Yisheng, Shanghai, China). Briefly, transfected GCs were lysed with the provided lysis buffer. The lysates were then subjected to sequential addition of the firefly luciferase assay reagent and Renilla luciferase assay reagent. Luminescence was measured using a luminometer, with the activity of firefly luciferase serving as the primary reporter and Renilla luciferase as the internal control. The relative luciferase activity was determined by calculating the ratio of firefly luminescence to Renilla luminescence.

### 2.12. Western Blot Analysis

The total proteins were extracted from GCs and follicles using RIPA lysis buffer (Thermo Scientific, Waltham, MA, USA), and the protein concentrations were determined using a BCA assay kit (BioSharp, Chengdu, China). The protein samples were separated by electrophoresis on 4–20% SDS polyacrylamide gel (Solabrio, Beijing, China). The separated proteins were then transferred to polyvinylidene fluoride (PVDF) membrane using the eBlotTM L1 membrane converter (GenScript, Nanjing, China). The PVDF membranes were blocked with 5% skim milk powder in Tris-buffered saline containing Tween-20 (TBST) for 2 h at room temperature, followed by incubated with diluted primary antibodies at 4 °C overnight. The primary antibody used included the following: anti-STAT4 (13028-1-AP, Affinity, 1:1000), anti-KISS1 (36939, Affinity, 1:1000), anti-GAPDH (10494-1-AP, Proteintech, 1:10,000), anti-α-Tubulin (AF7010, Affinity, 1:5000), anti-CCNE1 (AF4713, Affinity, 1:2000), anti- Caspase9 (AF6348, Affinity, 1:2000), anti-BIM (21280-1, Signalway, 1:1000), anti-p65 (10745-1-AP, Proteintech, 1:3000), anti-FSHR (AF5242, Affinity, 1:2000), anti-STAR (DF6192, Affinity, 1:2000), anti-Caspase8 (AF6442, Affinity, 1:2000), anti-CDK4 (DF6102, Affinity, 1:2000), anti-CCNB2 (bs-6656R, Affinity, 1:2000) and anti-CYP19A1 (40809, Affinity, 1:2000). After washing, the membranes were incubated with goat anti-rabbit IgG H&L (HRP) (abl50079, Abcam, 1:10,000) for 2 h at room temperature [35]. Protein bands were visualized using a BCL color kit and detected using the Odyssey Fc Imaging System (LI-COR Biosciences, Lincoln, NE, USA). The gray values of the protein bands were quantified using Image J 1 software.

### 2.13. Statistical Analysis

Statistical analysis was performed using GraphPad Prism 8.0 (GraphPad Software, Chicago, IL, USA). The experiment was set up with at least three biological replicates for each group. Significant differences were assessed using Student’s *t*-test, and all data were expressed as mean ± standard deviation (SD). Statistical significance was defined as *** *p* < 0.001, ** *p* < 0.01 and * *p* < 0.05.

## 3. Results

### 3.1. DNA Methylation May Regulate STAT4 to Increase Apoptosis in GCs

**Figure 1:**
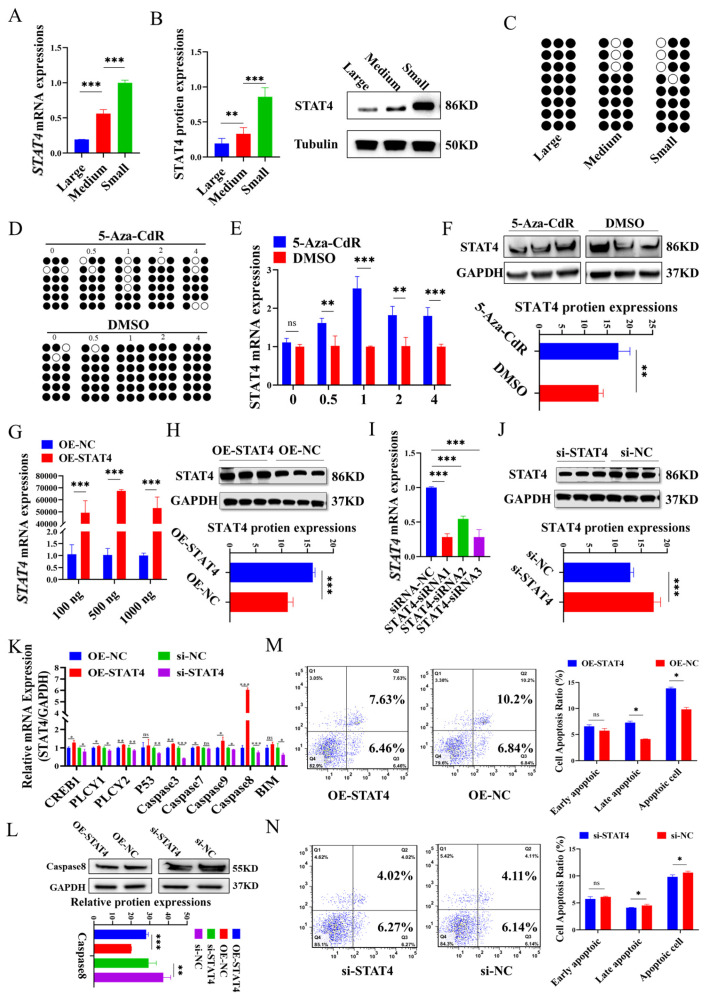
DNA methylation of *STAT4* elevates while the expression of *STAT4* decreases during the development of follicles. The mRNA (**A**) and protein (**B**) levels of *STAT4* in large (5–7 mm in diameter), medium (3–5 mm) and small (1–3 mm) follicles. (**C**) BSP showed the methylation status of *STAT4* in large (5–7 mm), medium (3–5 mm) and small (1–3 mm) follicles. (**D**) BSP assay showed the methylation status of *STAT4* in GCs treated with 5-Aza-CdR. The mRNA (**E**) and protein (**F**) levels of *STAT4* in GCs treated with 5-Aza-CdR. The mRNA (**G**) and protein (**H**) levels of *STAT4* in GCs transfected with *STAT4* overexpression plasmid. The mRNA (**I**) and protein (**J**) levels of *STAT4* after transfection with *STAT4*-siRNAs in GCs. (**K**) The mRNA levels of apoptosis-related genes with *STAT4* overexpression and knockdown. (**L**) The protein levels of Casp8 with *STAT4* overexpression and knockdown. The apoptosis rates in GCs with *STAT4* overexpression (**M**) and knockdown (**N**) assessed via flow cytometry. ns: not significant, *** *p* < 0.001, ** *p* < 0.01 and * *p* < 0.05.

The results indicated that both mRNA (Figure 1A) and protein (Figure 1B) expression of *STAT4* were significantly elevated in small follicles (1–3 mm) compared to medium (3–5 mm) and large follicles (5–7 mm). According to the CpG island prediction website (https://www.methprimer.com/), we found that the region −1220 bp/−1420 bp had more CG sites in the promoter of *STAT4*, including three specific CG sites (−1219 bp, −1256 bp and −1292 bp). The DNA methylation of *STAT4* was obviously increased in the large follicles when contrasted with small and medium follicles (Figure 1C). After treating with 5-Aza-CdR, it was found that 5-Aza-CdR could cause demethylation at the CG site of *STAT4*, and the effect was most significant at a concentration of 1 µM (Figure 1D). Moreover, the expression of *STAT4* also showed a similar trend after 5-Aza-CdR treatment, and the effect was most significant at a concentration of 1 µM (Figure 1E). Therefore, further methylation experiments were conducted, and the concentration of 1 µM 5-Aza-CdR was selected for subsequent experiments.

To investigate the impact of *STAT4* on apoptosis in GCs, *STAT4* overexpression plasmids and three *STAT4*-siRNAs (*STAT4*-siRNA1, *STAT4*-siRNA2 and *STAT4*-siRNA3) were transfected into GCs. The results showed a significant upregulation of both mRNA (Figure 1G) and protein levels (Figure 1H) of *STAT4* in GCs with *STAT4* overexpression (OE-*STAT4*) at a concentration of 500 ng/mL. In contrast, transfection with *STAT4*-siRNA1 remarkably reduced the mRNA (Figure 1I) and protein levels of *STAT4* (Figure 1J). Subsequently, we revealed that *STAT4* overexpression significantly enhanced the mRNA expressions of apoptosis-related genes, including *CREB1*, *PLCγ1*, *PLCγ2*, *Casp3*, *Casp8* and *Casp9* (Figure 1K), and remarkably upregulated the protein levels of *Casp8* (Figure 1L). Conversely, *STAT4* knockdown produced the opposite effects in GCs. In addition, OE-*STAT4* substantially elevated the apoptosis in GCs (Figure 1M), whereas this effect was significantly attenuated by *STAT4* knockdown (Figure 1N). These findings indicated that DNA hypomethylation may upregulate *STAT4* expression to induce apoptosis in GCs.

### 3.2. STAT4 Inhibits the Cellular Proliferation and E2 Secretion in GCs

**Figure 2:**
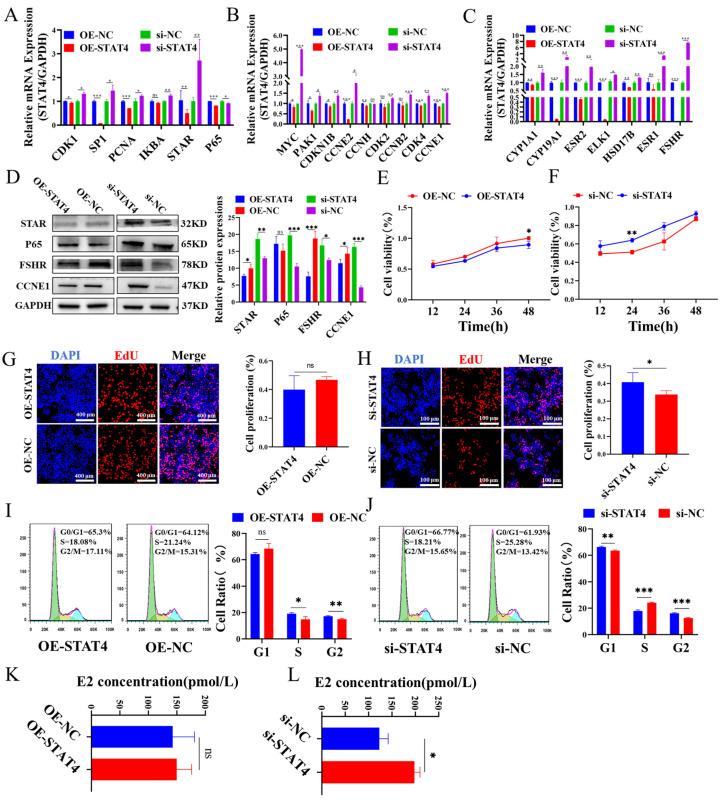
Effects of *STAT4* on the function of GCs. The mRNA of cellular proliferation (**A**), cell cycle (**B**) and estrogen secretion-related genes (**C**) detected in GCs with *STAT4* overexpression and knockdown. (**D**) The protein levels of STAR, P65, FSHR and CCNE1 in GCs with *STAT4* overexpression and knockdown. The CCK-8 assay shows the viability of GCs with *STAT4* overexpression (**E**) and knockdown (**F**). The EDU assay shows the proliferation of GCs with *STAT4* overexpression (**G**) and knockdown (**H**). The measurements of cell cycle distributions in GCs with *STAT4* overexpression (**I**) and knockdown (**J**). The measurements of E2 secretions in GCs with *STAT4* overexpression (**K**) and knockdown (**L**). ns: not significant, *** *p* < 0.001, ** *p* < 0.01 and * *p* < 0.05.

Subsequently, the effect of *STAT4* on the proliferation and E2 secretion in GCs was detected. Results showed that *STAT4* overexpression significantly reduced the mRNA levels of proliferation-related genes, including *CDK1*, *SP1*, *PCNA* and *STAR* (Figure 2A), as well as cell cycle-related genes such as *MYC*, *PAK1*, *CDKN1B*, *CCNE2*, *CDK4* and *CCNE1* (Figure 2B). Additionally, genes involved in estrogen biosynthesis such as *CYP19A1*, *ESR2* and *FSHR* were also markedly reduced (Figure 2C). Correspondingly, the protein levels of STAR, FSHR and CCNE1 were significantly suppressed by *STAT4* overexpression (Figure 2D). Conversely, *STAT4* knockdown notably promoted the expressions of genes associated with proliferation, cell cycle and estrogen secretion. Moreover, *STAT4* overexpression and knockdown notably restrained and enhanced the viability of GCs, respectively (Figure 2E,F). Correspondingly, *STAT4* overexpression inhibited GC proliferation (Figure 2G), whereas si-*STAT4* markedly enhanced it (Figure 2H). Meanwhile, *STAT4* overexpression significantly decreased the number of GCs that progressed to the S-phase (Figure 2I), while si-*STAT4* significantly prompted the progression of GCs into the S-phase (Figure 2J). Additionally, *STAT4* overexpression suppressed E2 secretion in GCs (Figure 2K), whereas si-*STAT4* exhibited the opposite effects (Figure 2L). In summary, *STAT4* acted as an inhibitor of GC proliferation, arrested cell cycle in the S phase and decreased E2 secretion.

### 3.3. DNA Hypomethylation of STAT4 Induces Apoptosis in GCs

**Figure 3:**
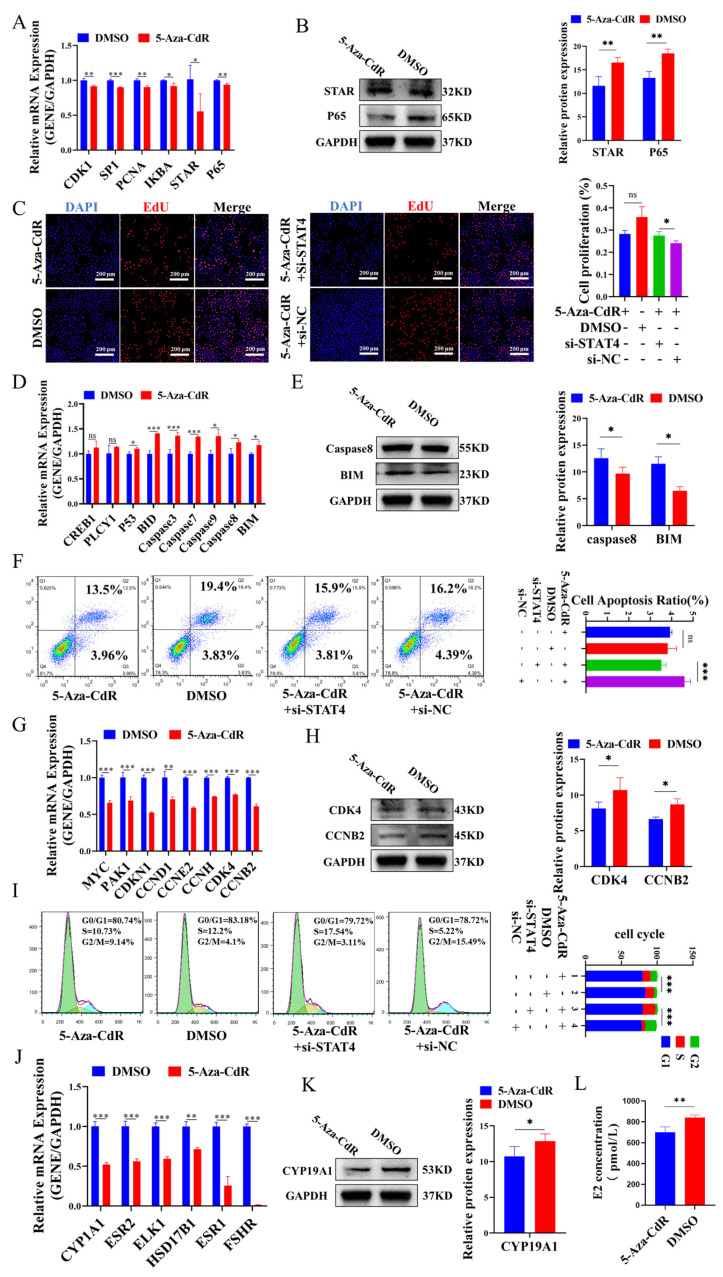
Changes in DNA methylation in *STAT4* promoter regulate the functions of GCs. The mRNA (**A**) and protein (**B**) levels of proliferation-related genes in GCs treated with 5-Aza-CdR. (**C**) EdU assay showing the proliferation rates of GCs with 5-Aza-CdR and si-*STAT4* treatments. (**D**,**E**) The mRNA (**D**) and protein (**E**) levels of apoptosis-related genes in GCs treated with 5-Aza-CdR. The apoptosis rates in GCs treated with 5-Aza-CdR and si-*STAT4* assessed via flow cytometry (**F**). The mRNA (**G**) and protein (**H**) levels of cell cycle-related genes in GCs treated with 5-Aza-CdR. (**I**) The cell cycle distribution of GCs treated with 5-Aza-CdR and si-*STAT4*. The mRNA (**J**) and protein (**K**) levels of estrogen secretion-related genes in GCs treated with 5-Aza-CdR. (**L**) The measurements of E2 secretions in GCs treated with 5-Aza-CdR. ns: not significant, *** *p* < 0.001, ** *p* < 0.01 and * *p* < 0.05.

To further explore the role of DNA hypomethylation of *STAT4* in regulating GC function, cellular apoptosis, proliferation, cell cycle and estrogen secretion were detected in GCs treated with 5-Aza-CdR. We revealed that 5-Aza-CdR significantly inhibited the mRNA of *CDK1*, *SP1*, *PCNA*, *IKBA* and *P65* (Figure 3A), as well as the protein expressions of STAR and P65 (Figure 3B). Correspondingly, 5-Aza-CdR reduced the proliferation of GCs, while co-treatment with 5-Aza-CdR and si-*STAT4* significantly restored their proliferation (Figure 3C). Additionally, we found that 5-Aza-CdR significantly enhanced the mRNA (Figure 3D) and protein levels (Figure 3E) of *Caspase8* and *BIM*, resulting in elevated GC apoptosis. However, co-treatment with 5-Aza-CdR and si-*STAT4* significantly decreased the apoptosis of GCs (Figure 3F). Moreover, 5-Aza-CdR markedly restrained the mRNA (Figure 3G) and protein levels (Figure 3H) of CDK4 and CCNB2 and notably reduced the number of GCs that progressed to the S-phase and increased the number of GCs that progressed to the G2/M-phase (Figure 3I), with opposite effects observed upon co-treatment with si-*STAT4*. Similarly, 5-Aza-CdR significantly downregulated the mRNA (Figure 3J) and protein levels (Figure 3K) of CYP19A1 and inhibited E2 secretion in GCs (Figure 3L). Taken together, our results suggested that DNA hypomethylation of *STAT4* enhanced apoptosis, inhibited cellular proliferation, arrested cell cycle progress and decreased E2 secretion in GCs.

### 3.4. STAT4 Induces Apoptosis in GCs While Inhibiting the Expression of KISS1

**Figure 4:**
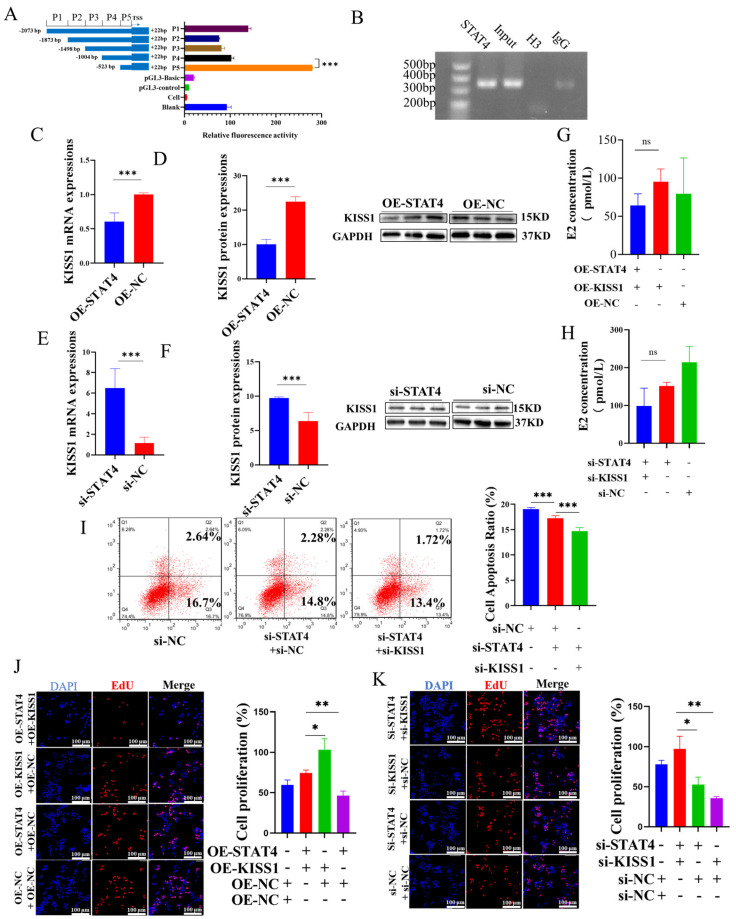
*STAT4* regulates the expression of *KISS1*, and thus promotes apoptosis in GCs. (**A**) Analysis of the activity of the *KISS1* promoter region specific segment via dual-luciferase reporter gene assay. (**B**) The ChIP experiment validates the binding of *STAT4* to the *KISS1* promoter region. Overexpression (**C**,**D**) and interference (**E**,**F**) of *STAT4* affected the mRNA and protein levels of *KISS1*. Effects of E2 in GCs with co-transfection of OE-*STAT4* and OE-*KISS1* (**G**), si-*STAT4* and si-*KISS1* (**H**) via ELISA. (**I**) The apoptosis rates with co-transfection of si-*STAT4* and si-*KISS1* via flow cytometry. (**J**) The effects of co-transfection of overexpression of *STAT4* and *KISS1* via EdU. (**K**) The effects of co-transfection with si-*STAT4* and si-*KISS1* on the proliferation of GCs. ns: not significant, *** *p* < 0.001, ** *p* < 0.01 and * *p* < 0.05.

Preliminary software prediction (https://tfbind.hgc.jp/) identified five potential *STAT4* binding sites within the *KISS1* promoter region, suggesting that *STAT4* might directly target *KISS1* to regulate its expression in GCs. To elucidate the molecular regulatory mechanism between *STAT4* and *KISS1*, recombinant plasmids containing five deletion fragments (P1: −2073/+22, P2: −1873/+22, P3: −1498/+22, P4: −1004/+22 and P5: −523/+22) of the *KISS1* promoter, along with the pGL3-TK internal reference, were sequentially transfected into GCs. We found that all fragments exhibited varying degrees of fluorescence activity, with the P5 fragment exhibiting the highest activity compared to the control. Using a pull-down assay with the *STAT4* antibody and the site vector (−1004/−523 bp), ChIP analysis confirmed significant binding of *STAT4* to this region within the *KISS1* promoter (−1004/−523 bp; Figure 4B). Subsequently, we found that the mRNA expression and protein levels of *KISS1* decreased (Figure 4C,D) when overexpression of *STAT4* occurred, and we obtained the opposite results (Figure 4E,F) when interference of *STAT4* occurred. Co-treatment of si-*STAT4* and si-*KISS1* significantly reduced GC apoptosis (Figure 4I) and reduced the E2 secretion, but not significantly (Figure 4H). Subsequently, OE-*KISS1* promoted GC proliferation, but simultaneous overexpression of both *KISS1* and *STAT4* (OE-*KISS1* and OE-*STAT4*) notably inhibited this proliferative effect (Figure 4J). Conversely, co-silencing of *STAT4* and *KISS1* yielded opposite outcomes (Figure 4K). These findings indicated that *STAT4* binds to the *KISS1* promoter, thereby suppressing *KISS1* expression and promoting apoptosis in GCs.

### 3.5. STAT4 Inhibits Follicular Development in Pigs

**Figure 5:**
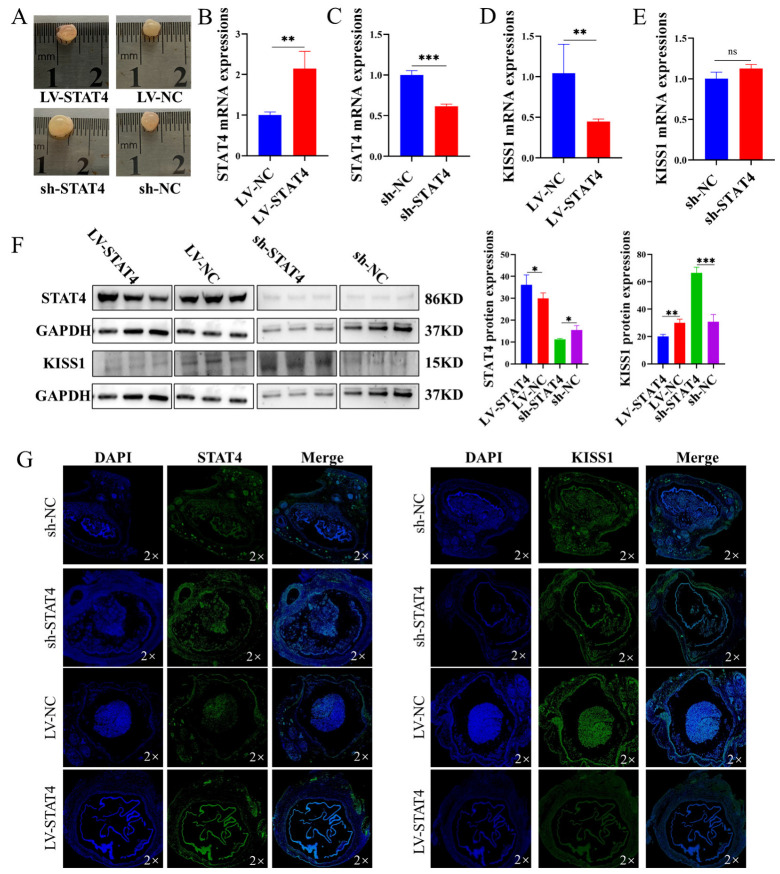
*STAT4* inhibits the development of porcine follicles. (**A**) Photos of porcine follicles cultured in vitro after transfection with LV-*STAT4* and sh-*STAT4* on the first and third days. (**B**,**C**) The efficiency of LV-*STAT4* (**B**) and sh-*STAT4* (**C**). (**D**,**E**) The efficiency of LV-*KISS1* (**D**) and sh-*KISS1* (**E**). (**F**) The protein levels of *STAT4* and *KISS1* in GCs were assessed after transfection with LV-*STAT4* and sh-*STAT4*. (**G**) The protein fluorescence intensities of *STAT4* and *KISS1* in follicles were assessed on the third day after LV-*STAT4* and sh-*STAT4* transduction via immunofluorescence. ns: not significant, *** *p* < 0.001, ** *p* < 0.01 and * *p* < 0.05.

To further investigate the biological function of *STAT4* in follicular development, lentiviral vectors for *STAT4* overexpression and knockdown (LV-*STAT4* and sh-*STAT4*, respectively), along with their respective negative controls (LV-NC and sh-NC, respectively), were constructed and transfected into porcine follicles cultured in vitro. Overexpression of *STAT4* led to the loss of follicular blood vessels and the opacity of follicular fluid (Figure 5A). Consistently, LV-*STAT4* significantly upregulated both the mRNA and protein levels of *STAT4* while significantly suppressing the mRNA and protein levels of *KISS1*. In contrast, sh-*STAT4* exhibited the opposite effects (Figure 5B–F). Immunofluorescence analysis further confirmed that sh-*STAT4* enhanced *KISS1* protein expression, whereas LV-*STAT4* suppressed *KISS1* protein in porcine follicles (Figure 5G). These results indicate that *STAT4* inhibited follicular development by repressing *KISS1* transcription.

### 3.6. STAT4 Blocks Follicular Development in Mice

**Figure 6:**
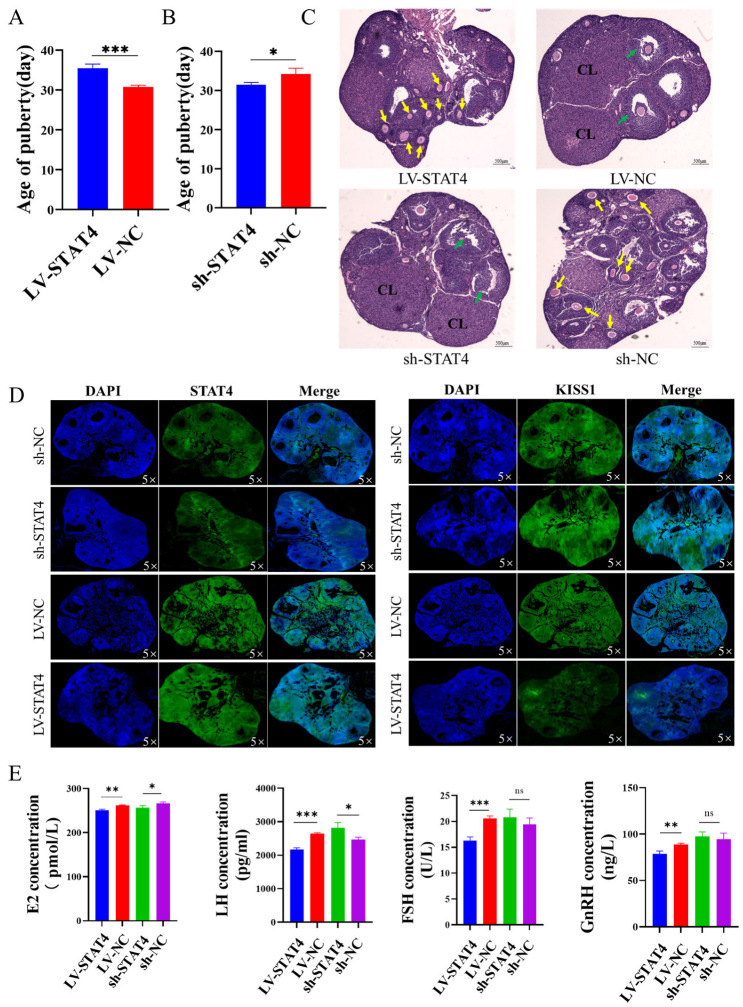
*STAT4* hinders follicular development in mice. (**A**,**B**) The effects of LV-*STAT4* (**A**) and sh-*STAT4* (**B**) on the initiation of puberty in mice. (**C**) Example of the ovaries from LV-*STAT4-* and sh-*STAT4*-injected mice collected at age 49 days and stained with HE. Yellow arrows indicate primordial follicles, green arrows indicate antral follicles, and CL points to an example of corpora lutea. (**D**) The protein fluorescence intensities of *STAT4* and *KISS1* in mice ovaries after LV-*STAT4* and sh-*STAT4* transduction via immunofluorescence. (**E**) The effects of LV-*STAT4* and sh-*STAT4* on the concentrations of E2, LH, FSH and GnRH in mice serum via ELISA. ns: not significant, *** *p* < 0.001, ** *p* < 0.01 and * *p* < 0.05.

To further verify the role of *KISS1* mediated by *STAT4* on follicular development, LV-*STAT4* and sh-*STAT4* were injected into the ovaries of 4-week-old C57BL/6 mice. It was found that LV-*STAT4* inhibited sexual maturity (Figure 6A), while sh-*STAT4* promoted sexual maturity in these mice (Figure 6B). Moreover, the antral follicles and corpus luteum were increased by sh-*STAT4*. Conversely, the results obtained with LV-*STAT4* were opposite to this observation (Figure 6C). Furthermore, immunofluorescence analysis of ovarian tissues revealed that sh-*STAT4* upregulated the protein levels of *KISS1*, whereas LV-*STAT4* downregulated them (Figure 6D). Additionally, the ELISA assay indicated that LV-*STAT4* significantly reduced the serum concentrations of key reproductive hormones, including E2, FSH, LH and GnRH (Figure 6E). These results indicate that *STAT4* inhibited follicular development and sexual maturity by suppressing the transcription of *KISS1*.

## 4. Discussion

Numerous studies have demonstrated that GCs undergo excessive apoptosis [36], leading to follicle atresia and impaired follicular development, which in turn leads to delayed sexual maturation and reduced reproductive performance in mammals [37,38], and the apoptosis of GCs is affected by many growth factors. For example, FSH promotes the proliferation and estradiol secretion of follicular cells by binding to granulosa cell receptors and upregulating the expressions of intracellular anti-apoptotic proteins XIAP and FLIP to inhibit apoptosis, thus promoting the survival and growth of follicular cells [39,40,41]. Notch signaling, an evolutionarily conserved pathway, is involved in ovarian follicle development by regulating the proliferation of GCs [42]. In the bovine estrous cycle, the apoptosis of GCs caused dominant follicle atresia during the non-ovulation period [43]. Research about follicular atresia in pigs indicated that during follicular development, apoptotic cell death was involved in the degeneration of GCs [44]. In this study, we found that overexpression of *STAT4* promoted GC apoptosis (Figure 1M) and hindered granulosa cell proliferation (Figure 2G), cyclic processes (Figure 2I) and estrogen secretion (Figure 2K). Meanwhile, overexpression of *STAT4* promoted mRNA expression and protein levels of genes related to the apoptosis pathway (*CREB1*, *PLCY1*, *PLCY2*, *P53*, *Casp3*, *Casp7*, *Casp9*, *Casp8*, *BIM*) (Figure 1K), inhibited the expression of genes related to cell proliferation (*CDK1*, *SP1*, *PCNA*, *IKBA*, *STAR*, *P65*) (Figure 2A), cyclic processes (*MYC*, *PAK1*, *CDKN1B*, *CCNH*, *CCNE2*, *CDK2*, *CCNB2*, *CDK4*, *CCNE1*) (Figure 2B) and estrogen secretion (*CYP1A1*, *CYP19A1*, *ESR2*, *ELK1*, *HSD17B*, *ESR1*, *FSHR*) (Figure 2C). These results show that *STAT4* promotes apoptosis and inhibits proliferation of GCs.

DNA methylation is a widely studied epigenetic modification that typically suppresses gene expression without altering the gene’s sequence by DNA methyltransferases, including DNMT1, DNMT3a and DNMT3b [45,46,47]. 5-Aza-CdR, a derivative of 2′-deoxycytidine, is a DNA methyltransferase inhibitor that substitutes cytosine in DNA molecules, reducing DNA methylation by inhibiting DNMTs’ activity [48]. Studies have indicated that the demethylation of the Lhcgr promoter region is a key mechanism regulating cell type-specific differentiation during follicular development [49]. In polycystic ovary syndrome, lnc-MAP3K13-7:1 inhibited the proliferation of ovarian GCs via DNMT1 downregulation mediated by CDKN1A promoter hypomethylation [50]. Moreover, in human T cells, the expression of *STAT4* is regulated by DNA methylation [51]. In patients with inflammatory bowel diseases (IBDs), DNA methylation of *STAT4* promoter is lower than in healthy individuals [52]. During mouse follicular development, large-scale DNA methylation occurs in proliferating ovarian GCs [53]. Another study showed that DNA promoter methylation is negatively correlated with lncRNA during puberty onset, and the methylation regulated the initiation of puberty via lncRNA [19]. In our study, we found that with the growth of follicles, the methylation of the *STAT4* promoter region gradually increases, but not significantly (Figure 1C), and the expression of *STAT4* gradually decreases (Figure 1A,B). Although the methylation status in small size follicles is not significantly different compared with medium size follicles, we consider that the expression of *STAT4* in follicles is regulated by multiple factors, such as histone acetylation [54] and DNA methylation [55]. DNA methylation changed the chromatin conformation and transcription factor binding, possibly. Otherwise, we found some transcription factors combined with the region −1220 bp/−1420 bp of the *STAT4* promoter by tfbind (https://tfbind.hgc.jp/), including SP1, GATA and P53, and these transcription factors played important roles in ovarian development [56]. Changes in DNA methylation might affect the binding of these transcription factors. For instance, changes in DNA methylation of the *STAT4* promoter disrupted the binding of SP1 and significantly reduced *STAT4* promoter activity in humans [51]. Knockdown p53 significantly reduced the expression of *STAT4* in mice tissue [57]. Moreover, in cows diagnosed with mastitis, the methylation of the *STAT5A* promoter was lowest compared with JAK2 and CD4, about 9–11%, but the expression of *STAT5A* still changed significantly [58]. Therefore, although the change in DNA methylation is small, it still significantly regulates *STAT4* expression. In the regulation of gene expression, small DNA methylation changes may also regulate *STAT4* expression by affecting chromatin structure or the transcription factor binding sites [59]. Our results showed that changes in the methylation status of the *STAT4* promoter may be one of the reasons for this regulation. Therefore, to further investigate whether the methylation status of the *STAT4* promoter region influences *STAT4* expression, we treated GCs with the methyltransferase inhibitor 5-Aza-CdR and analyzed the methylation status of the *STAT4* promoter region and *STAT4* expression levels. We found that at a concentration of 1 µM 5-Aza-CdR, the methylation level of the *STAT4* promoter region was significantly reduced, and *STAT4* expression was highest. These results suggest that changes in the methylation status of the *STAT4* promoter region may regulate *STAT4* expression levels (Figure 1D). The mRNA and protein expression of *STAT4* were detected when the content of 5-Aza-CdR was 1 μm (Figure 1C), and it was found that the mRNA and protein expression of *STAT4* were significantly decreased at this time (Figure 1E,F). Then, we co-treated GCs in vitro with 5-Aza-CdR and si-RNA and found that after co-treatment, apoptosis and related pathway genes of GCs were inhibited (Figure 3F), and the proliferation (Figure 3C), cycle process (Figure 3I) and expressions of related proteins of GCs were promoted.

Moreover, we explored the effect of *STAT4* on transcriptional regulation of *KISS1*. According to the information provided on the website (https://tfbind.hgc.jp/), *STAT4* is identified as a transcription factor for *KISS1*. Furthermore, the results of the dual-luciferase reporter gene assay demonstrated that *STAT4* binds to the promoter region −1004 bp/−523 bp of *KISS1*. On the other hand, the expression of *KISS1* was decreased when we overexpressed *STAT4* in GCs. Otherwise, there are numerous studies showing that *STAT4* regulates the transcription of *KISS1* [60,61]. The research showed that *STAT4* regulated the transcription of *KISS1* to inhibit the oxidative damage, inflammation and neuronal apoptosis in the model of Parkinson’s disease [60]. Our team has previously also discovered that *STAT4* acts as a transcription factor for *KISS1* using a different website [31]. In this study, we further investigated how changes in DNA methylation in the *STAT4* promoter region alter the expression of *KISS1* and enhance the apoptosis of GCs. Additionally, our findings reveal that the co-expression of *STAT4* and *KISS1* significantly influences apoptosis, proliferation, cell cycle progression and hormone secretion, as well as the underlying signaling pathways. To further investigate the effects of *STAT4* on ovarian follicle growth and development, we conducted in vitro follicle culture experiments (Figure 5) and in vivo mice studies (Figure 6). These results provide a foundation for further research into factors affecting follicular development in female mammals.

The primary regulatory pathway for the onset of puberty in mammals is the hypothalamic–pituitary–ovarian (HPO) axis [62]. E2 plays a dual role in the regulation of this HPO axis [63]. E2 promotes the release of GnRH and LH through *KISS1* neurons in the hypothalamus during puberty onset in mammals [64], which is the positive feedback mechanism. In our study, we hypothesized that *STAT4* affected E2 secretion by regulating the expression of *KISS1* (Figure 4G,H), and it might be the positive feedback to promote follicle development. As an important experimental animal model, C57BL/6 mice have been widely used in ovarian-related studies. Some studies have analyzed the levels of luteinizing hormone (LH), follicle-stimulating hormone (FSH) and prolactin in the plasma and pituitary of mice at different ages and stages of the estrous cycle [65]. Additionally, age-related changes in estrogen receptor expression have been observed in middle-aged mice, independent of estrous cycle status, suggesting that the regulation of estrogen receptors may influence follicular development [66]. In this experiment, C57BL/6 mice were infected with LV-*STAT4* and sh-*STAT4* and their control group with LV-NC and sh-NC. After the treatment with *STAT4*, the degree of apoptosis of GCs in follicles increased (Figure 6C). Then, the estrus of mice, the levels of serum hormones GnRH, FSH, LH and E2 and the expression of *KISS1* were measured. It was found that, compared with the control group, LV-*STAT4* inhibited the estrus of mice, and the levels of serum hormones GnRH, FSH, LH and E2 decreased. sh-*STAT4* promoted estrus in mice, and the levels of GnRH, FSH, LH and E2 in serum increased, indicating that the *STAT4* gene could inhibit estrus in mice. Otherwise, we also found that LV-*STAT4* decreased blood vessels in the follicles of pigs (Figure 5A) and suppressed *KISS1* protein in porcine follicles (Figure 5G). Above all, we hypothesized that *STAT4* may regulate *KISS1* transcription, thereby influencing estrogen secretion by GCs in sow follicles and subsequently affecting follicular development. However, this study had some limitations. On the one hand, due to the post-translational modification, alternative splicing and proteolytic cleavage of *KISS1* and the *KISS1* proteins being short peptides that are easily degraded, these may lead to extra bands. On the other hand, the change in DNA methylation induced by 5-Aza-CdR is small in −1220 bp/−1420 bp of the *STAT4* promoter, but the expression of *STAT4* is significant. We guess that 5-Aza-CdR changes the methylation of the other expected region −1220 bp/−1420 bp in the *STAT4* promoter. The research showed that the change in DNA methylation in −2225 bp/+605 bp of the *STAT4* promoter significantly reduced *STAT4* promoter activity in humans [51]. In subsequent research, we will try to use more accurate experimental methods, such as CRISPR/dCas9, to change the DNA methylation of specific CG sites in the *STAT4* promoter and investigate the influence of DNA methylation on *STAT4* expression.

## 5. Conclusions

Based on the above findings, it is evident that in normally developing ovarian follicles, methylation of the *STAT4* promoter in mature follicles is significantly higher compared to immature follicles. *STAT4* inhibits the expression of *KISS1*, induces apoptosis of GCs and suppresses both proliferation and estrogen secretion of GCs. Moreover, the results showed that *STAT4* inhibits follicular development, sexual maturation and hormone secretion in mice by suppressing the transcription of *KISS1*. Therefore, we speculate that DNA methylation regulates the transcription of *STAT4*, thereby promoting *KISS1* expression, fostering follicular development and ultimately facilitating puberty onset in female mammals.

## Data Availability

The data underlying this research will be provided by the corresponding authors upon reasonable request.

## Abbreviations

The following abbreviations are used in this manuscript:

STAT4	Signal transducer and activator of transcription 4
KISS1	Kisspeptin-1
GCs	Granulosa cells
5-Aza-CdR	5-azacytidine
E2	Estradiol
LH	Luteinizing hormone
FSH	Follicle-stimulating hormone
GnRH	Gonadotropin-releasing hormone
